# Parental overprotection engenders dysfunctional attitudes about achievement and dependency in a gender-specific manner

**DOI:** 10.1186/1471-244X-13-345

**Published:** 2013-12-24

**Authors:** Koichi Otani, Akihito Suzuki, Yoshihiko Matsumoto, Naoshi Shibuya, Ryoichi Sadahiro, Masanori Enokido

**Affiliations:** 1 Department of Psychiatry, Yamagata University School of Medicine, 2-2-2 Iidanishi, Yamagata 990-9585, Japan

**Keywords:** Dysfunctional attitude, DAS-24, Parenting, PBI

## Abstract

**Background:**

It has been suggested that dysfunctional attitudes, cognitive vulnerability to depression, have developmental origins. The present study examined the effects of parental rearing on dysfunctional attitudes in three areas of life with special attention to gender specificity.

**Methods:**

The subjects were 665 Japanese healthy volunteers. Dysfunctional attitudes were assessed by the 24-item Dysfunctional Attitude Scale, which has the Achievement, Dependency and Self-control subscales. Perceived parental rearing was assessed by the Parental Bonding Instrument, which has the Care and Protection subscales.

**Results:**

Higher scores of the Achievement (β = 0.293, *p < 0.01*) and Dependency (β = 0.224, *p < 0.05*) subscales were correlated with higher scores of the Protection subscale in the combination of mother and daughter, but not in other combinations of parents and recipients. Scores of the Self-control subscale were not correlated with paternal or maternal rearing scores.

**Conclusions:**

The present study suggests that parental overprotection engenders dysfunctional attitudes about achievement and dependency in a gender-specific manner.

## Background

Beck’s cognitive theory of depression [1,2] postulates that negative beliefs about one’s self and personal world formed by negative childhood experiences, especially with parents, predispose to depression. Once these maladaptive self-schemas are activated by life stressors, cognitive errors and negative automatic thoughts ensue, resulting in negative evaluations and interpretations that characterize clinical depression. Weissman and Beck [3] developed the Dysfunctional Attitude Scale (DAS) to measure these maladaptive self-schemas.

Subsequently, Power et al. [4] classified dysfunctional attitudes into three areas of life, i.e., achievement, dependency and self-control, and developed a 24-item version of the DAS (DAS-24) with corresponding three subscales. The Achievement subscale contains 8 items about achievement and failure, e.g., “If I fail partly, it is as bad as being a complete failure”. The Dependency subscale contains 8 items about dependency and approval, e.g., “I am nothing if a person I love doesn’t love me”. The Self-control subscale contains 8 items about necessity for self-control, e.g., “A person should do well at everything he undertakes”. Respondents rate the degree to which they match each phrase on a 1- to 7-point scale, where 1 is “totally disagree” and 7 is “totally agree”. Power et al. [4] proposed that usage of the three subscales is more useful than global measure to evaluate cognitive vulnerability, since individuals may have dysfunctional attitudes not in all areas but in one or two highly valued areas. Our recent study [5] showed that all subscales had negative correlations with the Self-directedness dimension of the Temperament and Character Inventory (TCI) [6], but they had different correlation patterns with other TCI dimensions, supporting the content specificity of the three subscales.

Under the influence of Bowlby’s attachment theory [7,8], Parker et al. [9] developed the Parental Bonding Instrument (PBI) with the Care and Protection subscales to assess attitudes and behaviors of parents. The Care subscale consists of 12 items, e.g., “Spoke to me with a warm and friendly voice”. The Protection subscale consists of 13 items, e.g., “Tried to control everything I did”. Subjects rate the degree to which their fathers or mothers match each phrase on a 0- to 3-point scale, where 0 is “very unlike” and 3 is “very like”. Higher scores of the Care subscale mean care and involvement, while lower scores of this subscale mean indifference and rejection. Higher scores of the Protection subscale mean control, overprotection and intrusion, while lower scores of this subscale mean encouragement of independence and autonomy. The PBI has been widely used in the field of developmental psychiatry, e.g., studies on the effects of parental rearing on depression-prone personality [10,11].

A few studies have examined the effects of parental rearing assessed by the PBI on formation of dysfunctional attitudes. Whisman and Kwon [12] reported that lower care of mothers was correlated with higher DAS scores in 150 undergraduates. Randolf and Dykman [13] reported that lower care and higher protection by both parents were related to higher DAS scores in 246 college students. These studies support Beck’s hypothesis that dysfunctional attitudes have developmental origins [1,2], but it remains to be elucidated what type(s) of dysfunctional attitudes is most likely to be influenced by parental rearing. Also, it is regrettable that in these studies males and females were not analyzed separately, since our previous studies showed gender specific effects of dysfunctional parenting on depression-prone personality [10,11].

Therefore, in the present study we examined the effects of parental rearing assessed by the PBI on dysfunctional attitudes in the three areas of life assessed by the DAS-24 with special attention to gender specificity.

## Methods

Originally, 713 physically healthy Japanese were recruited from medical students and hospital staffs living in Yamagata Prefecture. Psychiatric screening was conducted by brief interviews by well-trained psychiatrists and a questionnaire on present or past history of psychiatric disorders. Six items selected from the Structured Clinical Interview for DSM-IV Axis I Disorders [14] were used for the psychiatric interview. They were A1 for major depressive episode, A16 for manic episode, B1 for delusions, B6 for hallucinations, E2 for alcohol abuse and F68 for anxiety disorders. Out of the 713 cases, 14 had psychiatric disorders, 8 had parents divorced or deceased before the age of 16, and 26 had missing data. These 48 cases were excluded, and the remaining 665 cases were used for analyses. Four hundred forty-five were males, and 220 were females. The mean ± SD of age was 30.2 ± 9.4 years. The study protocol was approved by the Ethics Committee of Yamagata University School of Medicine, and all subjects provided written informed consent to participate.

Dysfunctional attitudes were measured by the Japanese version of the DAS-24 [15]. This Japanese version has been shown to have high reliability and validity [15]. In the present sample, Cronbach’s alphas for the Achievement, Dependency and Self-control subscales were 0.78, 0.75 and 0.60, respectively.

Perceived rearing attitudes and behaviors of parents during the first 16 years were assessed by the Japanese version of the PBI [16]. In the present sample, Cronbach’s alphas for the Paternal Care, Paternal Protection, Maternal Care and Maternal Protection subscales were 0.92, 0.84, 0.90 and 0.85, respectively.

Statistical analyses were performed by Student’s t test, linear regression analysis and multiple regression analysis, using SPSS 14.0 J for Windows (SPSS Japan Inc, Tokyo, Japan). After Bonferroni’s correction for multiple testing, a p value of less than 0.05 was considered statistically significant.

## Results

Table 1 shows the DAS-24 and PBI scores of the subjects. Table 2 shows the correlations among the DAS-24 scores, PBI scores and age. Tables 3 and 4 show the results of the multiple regression analyses of the DAS-24 scores with PBI scores and age in males and females, respectively. In males (Table 3), none of the DAS-24 subscale scores were correlated with the paternal rearing or maternal rearing scores. The scores of the Achievement (*p < 0.01*) and Dependency (*p < 0.01*) subscales were negatively correlated with age. In females (Table 4), higher scores of the Achievement (*p < 0.01*) and Dependency (*p < 0.05*) subscales were correlated with higher scores of the Protection subscale of mother, but not father. The scores of the Self-control subscale were not correlated with the paternal or maternal rearing scores.

**Table 1:** DAS-24 and PBI scores of subjects

	**Males**	**Females**
DAS-24		
Achievement	27.2 ± 7.7	25.9 ± 8.8
Dependency	31.8 ± 7.2	32.4 ± 7.1
Self-control	30.3 ± 6.3	28.6 ± 6.7**
Paternal		
Care	23.5 ± 6.8	24.3 ± 7.5
Protection	10.4 ± 5.4	10.4 ± 5.2
Maternal		
Care	28.1 ± 5.2	29.3 ± 5.8
Protection	11.4 ± 6.3	11.6 ± 6.9

Figures in the table show means ± SD.

*P* values are corrected by Bonferroni’s method for multiple testing.

**p < 0.01.

DAS-24 indicates 24-item Dysfunctional Attitude Scale; PBI, Parental Bonding Instrument.

**Table 2:** Correlations among DAS-24 scores, PBI scores and age in males (above diagonal) and females (below diagonal)

	**1**	**2**	**3**	**4**	**5**	**6**	**7**	**8**
1. Achievement	-	0.388*	0.657*	−0.056	0.097	−0.062	0.096	−0.143
2. Dependency	0.584*	-	0.333*	−0.007	0.042	0.053	0.073	−0.153*
3. Self-control	0.758*	0.506*	-	−0.008	−0.006	0.058	0.015	−0.084
4. Paternal care	0.065	0.220*	0.097	-	−0.410*	0.519*	−0.281*	−0.035
5. Paternal protection	0.119	−0.002	0.054	−0.270*	-	−0.336*	0.606*	0.025
6. Maternal care	−0.048	0.169	0.004	0.563*	−0.381*	-	−0.444*	−0.045
7. Maternal protection	0.255*	0.109	0.135	−0.201	0.614*	−0.426*	-	−0.064
8. Age	−0.076	−0.139	−0.064	−0.227*	0.039	−0.232*	−0.071	-

Figures in the table show r.

*P* values are corrected by Bonferroni’s method for multiple testing.

*p < 0.05.

DAS-24 indicates 24-item Dysfunctional Attitude Scale, PBI: Parental Bonding Instrument.

**Table 3:** Multiple regression analyses of DAS-24 scores with PBI scores and age in males

	**Achievement**	**Dependency**	**Self-control**
Paternal			
Care	−0.011	−0.040	−0.059
Protection	0.068	0.008	−0.029
Maternal			
Care	−0.026	0.113	0.100
Protection	0.031	0.098	0.055
Age	−0.144**	−0.144**	−0.077
Correlation coefficient	0.180*	0.189*	0.117

Figures in the table show β.

*P* values are corrected by Bonferroni’s method for multiple testing.

*p < 0.05; **p < 0.01.

DAS-24 indicates 24-item Dysfunctional Attitude Scale; PBI, Parental Bonding Instrument.

**Table 4:** Multiple regression analyses of DAS-24 scores with PBI scores and age in females

	**Achievement**	**Dependency**	**Self-control**
Paternal			
Care	0.111	0.159	0.124
Protection	−0.032	−0.038	−0.015
Maternal			
Care	−0.004	0.149	−0.007
Protection	0.293**	0.224*	0.165
Age	−0.029	−0.051	−0.025
Correlation coefficient	0.284**	0.303**	0.187

Figures in the table show β.

*P* values are corrected by Bonferroni’s method for multiple testing.

*p < 0.05; **p < 0.01.

DAS-24 indicates 24-item Dysfunctional Attitude Scale; PBI, Parental Bonding Instrument.

## Discussion

In the present study, parental overprotection was associated with increased dysfunctional attitudes in the areas of achievement and dependency. According to Bowlby’s attachment theory [7,8], the crucial roles of parents are to respond to a child’s desire for care and to encourage a child to explore the world, developing secure attachment in the child. The securely attached child builds up a self-model as being able to help oneself and as worthy of being helped should difficulties arise. In contrast, lack of care or overprotection of parents creates insecure attachment in a child. The insecurely attached child forms a self-model as being helpless and unworthy. This negative self-model tends to persist relatively unchanged, and leads to various psychopathologies such as depression in adulthood. On the other hand, cognitive theory of depression postulates that helplessness and unworthiness are the two major core beliefs organized in maladaptive self-schemas predisposing to depression [1,2]. In line with this, our recent study on the relationship of the DAS-24 with the TCI [5] showed that dysfunctional attitudes are closely connected with low self-directedness characterized by purposelessness, helplessness and low self-esteem [6]. Therefore, it is suggested that parental overprotection engenders insecure attachment (from the perspective of attachment theory) and dysfunctional attitudes (from the perspective of cognitive theory), both of which are characterized by an image of the self as helpless and unworthy. High persistence, i.e., a high level of perseveration and repetitive behaviors [6], related to dysfunctional attitudes about achievement [5], may represent a compensatory mechanism for helplessness and low self-esteem. Meanwhile, high reward dependence related to dysfunctional attitudes about dependency [5], may represent dependency on others to maintain a positive self-regard.

The limitation of the harmful effects of parental overprotection to the mother-daughter combination is in line with our previous result [11] on sociotropy, a personality vulnerability factor in cognitive theory of depression [1,2]. In a similar vein, we found that in women interpersonal sensitivity, a depression-prone personality trait closely related to insecure attachment [17], was increased only by maternal overprotection [10]. Taken together, it is possible that the link between dysfunctional parenting and cognitive vulnerability to depression or depression-prone personality is strongest in this combination. In the majority of cases a mother is usually the primary caregiver, and for a daughter a mother is the identification figure after the oedipal stage [18]. Therefore, in daughters harmful effects of anomalous maternal rearing may continue long and be condensed. On the other hand, in sons harmful effects of anomalous maternal rearing at early stages, if any, may weaken if paternal rearing at later stages is optimal. Incidentally, the decreases in dysfunctional attitudes about achievement and dependency according to age progress in males may be due to maturation effects.

In our recent study dysfunctional attitudes about self-control were associated with low harm avoidance, i.e., optimism and confidence [6], and we raised the possibility that extreme attitudes about self-control are not that dysfunctional when not activated or not dysfunctional at all [5]. The present result suggesting that only this group of dysfunctional attitudes lacks influence of anomalous parenting may partly support this view. Alternatively, the possibility that parental behaviors not covered by the PBI such as perfectionistic expectations and criticalness [13] engender extreme attitudes about self-control cannot be excluded.

There are some limitations in this study. Firstly, the psychiatric screening used might not be sufficient to exclude subjects with present or past history of psychiatric disorders. Secondly, as mentioned above parental behaviors not covered by the PBI may engender dysfunctional attitudes about self-control. Thirdly, as the subjects of this study were all Japanese medical students and hospital staffs, it may be risky to generalize the present results to other ethnic groups or general populations.

## Conclusions

The present study suggests that parental overprotection engenders dysfunctional attitudes about achievement and dependency in a gender-specific manner.

## Competing interests

All authors declare that they have no conflicts of interest.

## Authors’ contributions

KO conceptualized and designed the study, collected and interpreted the data, and drafted the manuscript. AS designed the study, collected and analyzed the data, and modified the manuscript. YM, NS, RS and ME collected the data. All authors read and approved the final manuscript.

## Pre-publication history

The pre-publication history for this paper can be accessed here:

http://www.biomedcentral.com/1471-244X/13/345/prepub
